# Supplementary material for: Codon Usage Bias Analysis in the Chloroplast Genome of Actinostemma tenerum (Cucurbitaceae)
Source: Curr Issues Mol Biol. 2025 Oct 10;47(10):833. doi: 10.3390/cimb47100833 (PMC12563308; doi:10.3390/cimb47100833)
Supplement: Supplementary file 1 [file cimb-47-00833-s001.zip › cimb-3886722-supplementary.pdf]

Supplementary files

Tables legends

Table S1. List of gene contents in *A. tenerum*.

Table S2. Basic parameters of codon usage bias of A. tenerum chloroplast genes.

Table S3. RSCU values reflecting codon usage bias in the chloroplast genes of *A. tenerum*.

Table S4. The quadrant distribution data of each gene in the PR2-plot in the chloroplast genes of *A. tenerum*.

Table S1. List of gene contents in *A. tenerum*.

Category	Groups of Genes	Names of Genes
Self-replication	Ribosomal RNA	*rrn4.5* *, *rrn5* *, *rrn16* *, *rrn23* *
	Transfer RNA	*trnA-UGC* *,†, *trnC-GCA*, *trnD-GUC*, *trnE-UUC*, *trnF-GAA*, *trnM-CAU* *, *trnG-GCC*, *trnG-UCC* †, *trnH-GUG*, *trnI-CAU* *, *trnI-GAU* *,†, *trnK-UUU* †, *trnL-CAA* *, *trnL-UAA* †, *trnL-UAG*, *trnN-GUU* *, *trnP-UGG*, *trnQ-UUG*, *trnR-ACG* *, *trnR-UCU*, *trnS-GCU*, *trnS-GGA*, *trnS-UGA*, *trnT-GGU*, *trnT-UGU*, *trnV-GAC**, *trnV-UAC* †, *trnW-CCA*, *trnY-GUA*,
	Small subunit of ribosome	*rps2*, *rps3*, *rps4*, *rps7* *, *rps8*, *rps11*, *rps12* *,†, *rps14*, *rps15*, *rps16* †, *rps18*, *rps19*
	Large subunit of ribosome	*rpl2* *,†, *rpl14*, *rpl16* †, *rpl20*, *rpl22*, *rpl23**, *rpl32*, *rpl33*, *rpl36*
	RNA polymerase subunit	*rpoA*, *rpoB*, *rpoC1* †, *rpoC2*
Photosynthesis	ATP synthase	*atpA*, *atpB*, *atpE*, *atpF* †, *atpH*, *atpI*
	NADH dehydrogenase	*ndhA* †, *ndhB* *,†, *ndhC*, *ndhD* *, *ndhE*, *ndhF*, *ndhG*, *ndhH*, *ndhI*, *ndhJ*, *ndhK*
	Cytochrome b/f complex	*petA*, *petB* †, *petD* †, *petG*, *petL*, *petN*
	Photosystem Ⅰ	*psaA*, *psaB*, *psaC*, *psaI*, *psaJ*
	Photosystem Ⅱ	*psbA*, *psbB*, *psbC*, *psbD*, *psbE*, *psbF*, *psbH*, *psbI*, *psbJ*, *psbK*, *psbL*, *psbM*, *psbN*, *psbT*, *psbZ*
	Rubisco large subunit	*rbcL*
Others	Proteolysis	*clpP*†
	Cytochrome *c* biogenesis protein	*ccsA*
	Acetyl-CoA carboxylase	*accD*
	Chloroplast envelope membrane protein	*cemA*
	maturase	*matK*
Unknown	Hypothetical reading frame	*ycf1*, *ycf2* *, *ycf3* †, *ycf4*

*, duplicated gene in IR; †, gene including one or two introns

Table S2. Basic parameters of codon usage bias of *A. tenerum* chloroplast genes.

Genes	Codon No.	GC1	GC2	GC3	GC_all	ENC	$GC3_s$
accD	492	38.82	37.60	28.86	35.09	43.84	28.5
atpA	508	54.72	40.16	25.59	40.16	46.22	25.6
atpB	499	55.71	41.68	28.66	42.02	46.19	29.4
atpE	135	51.11	37.78	31.11	40.00	50.61	31.8
atpF	185	46.49	30.27	30.81	35.86	44.43	30.6
atpI	248	50.00	36.69	23.79	36.83	45.00	23.8
ccsA	313	33.87	37.38	24.6	31.95	44.29	24.5
cemA	230	39.13	27.83	32.61	33.19	42.81	32.6
clpP	197	58.88	37.06	29.95	41.96	49.39	29.3
matK	505	39.41	31.68	28.32	33.14	47.79	27.9
ndhA	364	43.13	36.81	20.88	33.61	42.50	21.3
ndhB	511	41.88	39.14	30.92	37.31	46.90	31.1
ndhC	121	47.11	31.40	23.14	33.88	44.68	22.7
ndhE	101	40.59	33.66	26.73	33.66	49.58	26.3
ndhF	746	34.72	35.39	23.59	31.23	43.44	23.6
ndhG	177	41.81	35.59	28.25	35.22	49.72	28.6
ndhH	394	50.51	36.04	28.17	38.24	49.74	29.1
ndhI	173	43.35	35.26	23.12	33.91	47.53	22.9
ndhJ	159	49.69	37.11	28.30	38.36	39.32	28.1
ndhK	229	41.92	43.23	26.64	37.26	45.90	26.7
petA	321	53.89	35.51	30.22	39.88	49.01	30.0
petB	216	49.07	41.67	31.48	40.74	45.54	31.2
petD	161	51.55	39.13	27.33	39.34	37.25	27.7
psaA	751	51.93	43.54	32.89	42.79	48.08	32.9
psaB	735	49.12	43.13	28.71	40.32	44.84	28.9
psbA	354	50.00	42.94	31.64	41.53	39.38	31.7
psbB	509	54.62	46.17	28.29	43.03	43.64	28.5
psbC	474	53.59	45.99	30.8	43.46	42.41	30.5
psbD	354	52.26	43.22	31.92	42.47	44.14	31.8
rbcL	476	58.61	43.70	28.57	43.63	46.72	29.1
rpl14	123	52.85	37.40	21.14	37.13	41.43	21.8
rpl16	136	48.53	52.94	26.47	42.65	38.55	28.0
rpl2	275	50.91	49.09	30.18	43.39	49.36	31.2
rpl20	118	34.75	40.68	24.58	33.33	43.93	23.6
rpl22	167	37.72	35.93	30.54	34.73	49.89	31.5
rpoA	325	44.00	31.38	25.54	33.64	45.94	25.0
rpoB	1072	50.28	38.43	27.33	38.68	46.69	27.6
rpoC1	684	50.58	37.87	27.49	38.65	47.49	27.9
rpoC2	1433	44.94	37.75	27.08	36.59	47.17	26.9
rps11	139	51.80	57.55	28.78	46.04	46.67	29.5
rps12	124	51.61	47.58	31.45	43.55	47.29	32.2
rps14	101	45.54	47.52	31.68	41.58	36.24	31.6

rps18	102	37.25	42.16	25.49	34.97	44.21	25.8
rps2	237	45.15	43.88	28.69	39.24	47.27	28.0
rps3	219	47.03	33.33	21.00	33.79	42.89	21.1
rps4	202	50.50	37.13	24.26	37.29	48.06	24.4
rps7	156	53.21	44.87	23.08	40.38	42.91	23.7
rps8	135	40.00	40.74	27.41	36.05	35.34	27.3
ycf2	2284	41.64	34.24	36.56	37.48	50.54	36.5
ycf3	169	47.34	38.46	32.54	39.45	56.22	32.3
ycf4	185	41.08	40.00	36.76	39.28	55.19	36.7
Average	373	46.95	39.52	28.12	38.20	45.57	28.22

Table S3. RSCU values reflecting codon usage bias in the chloroplast genes of *A. tenerum*.

Symbol	Codon	No.	RSCU	Symbol	Codon	No.	RSCU
Ter	UAG	7	0.4119	Lys	AAA	706	1.5584
	UAA	34	2.0001		AAG	200	0.4416
	UGA	10	0.5883	Asn	AAU	691	1.5704
Ala	GCU	515	1.8952		AAC	189	0.4296
	GCC	160	0.5888	Pro	CCG	100	0.4964
	GCG	111	0.4084		CCA	220	1.092
	GCA	301	1.1076		CCU	320	1.588
Cys	UGU	166	1.4822		CCC	166	0.824
	UGC	58	0.5178	Gln	CAA	523	1.5382
Asp	GAC	146	0.3832		CAG	157	0.4618
	GAU	616	1.6168	Arg	CGC	79	0.417
Glu	GAG	251	0.5014		CGG	74	0.3906
	GAA	750	1.4986		CGA	271	1.4298
Phe	UUC	356	0.671		CGU	264	1.3932
	UUU	705	1.329		AGG	122	0.6438
Gly	GGG	234	0.676		AGA	327	1.7256
	GGA	535	1.5452	Ser	UCA	299	1.2582
	GGC	129	0.3724		UCG	120	0.5052
	GGU	487	1.4064		UCU	391	1.6452
His	CAC	108	0.4606		AGU	302	1.2708
	CAU	361	1.5394		UCC	232	0.9762
Ile	AUU	818	1.5075		AGC	82	0.345
	AUC	314	0.5787	Thr	ACG	102	0.424
	AUA	496	0.9141		ACA	284	1.1808
Leu	CUC	119	0.3588		ACU	393	1.634
	CUG	129	0.3888		ACC	183	0.7608
	CUA	271	0.8166	Val	GUU	388	1.4424
	CUU	424	1.278		GUC	125	0.4648
	UUG	409	1.2324		GUG	143	0.5316
	UUA	639	1.9254		GUA	420	1.5612
Met	AUG	433	1	Tyr	UAC	141	0.3956
Trp	UGG	346	1		UAU	572	1.6044

Table S4. The quadrant distribution data of each gene in the PR2-plot in the chloroplast genes of *A. tenerum*.

Genes	G3/(G3+C3)	A3/(A3+T3)	Genes	G3/(G3+C3)	A3/(A3+T3)
accD	0.53	0.36	*psbB*	0.54	0.4
atpA	0.52	0.49	*psbC*	0.49	0.41
atpB	0.5	0.51	*psbD*	0.44	0.37
atpE	0.62	0.48	*rbcL*	0.48	0.4
atpF	0.61	0.52	*rpl14*	0.46	0.53
atpI	0.37	0.44	*rpl16*	0.44	0.61
ccsA	0.53	0.43	*rpl2*	0.48	0.51
cemA	0.44	0.41	*rpl20*	0.59	0.47
clpP	0.51	0.51	*rpl22*	0.47	0.57
matK	0.55	0.42	*rpoA*	0.57	0.5
ndhA	0.47	0.5	*rpoB*	0.62	0.51
ndhB	0.43	0.46	*rpoC1*	0.55	0.47
ndhC	0.68	0.44	*rpoC2*	0.53	0.49
ndhE	0.48	0.35	*rps11*	0.47	0.51
ndhF	0.62	0.39	*rps12*	0.46	0.54
ndhG	0.6	0.39	*rps14*	0.66	0.61
ndhH	0.59	0.48	*rps18*	0.58	0.49
ndhI	0.53	0.41	*rps2*	0.68	0.44
ndhJ	0.69	0.42	*rps3*	0.52	0.59
ndhK	0.49	0.46	*rps4*	0.49	0.49
petA	0.52	0.42	*rps7*	0.53	0.58
petB	0.62	0.39	*rps8*	0.51	0.5
petD	0.55	0.48	*ycf2*	0.54	0.44
psaA	0.51	0.4	*ycf3*	0.56	0.47
psaB	0.57	0.38	*ycf4*	0.5	0.43
psbA	0.37	0.33			
